# The lytic *Myoviridae* of *Enterobacteriaceae* form tight recombining assemblages separated by discontinuities in genome average nucleotide identity and lateral gene flow

**DOI:** 10.1099/mgen.0.000169

**Published:** 2018-03-27

**Authors:** Tomaž Accetto, Nika Janež

**Affiliations:** ^1^​ Biotechnical Faculty, Animal Science Department, University of Ljubljana, Domžale, Slovenia; ^2^​ Center of Excellence for Biosensors Instrumentation and Process Control, Center for Biotechnology, Tovarniška 26, 5270 Ajdovščina, Slovenia

**Keywords:** *Myoviridae*, lytic, average nucleotide identity, evolution, recombination

## Abstract

In *Bacteria*, a working consensus of species circumscription may have been reached and one of the most prominent criteria is high average nucleotide identity (ANI). ANI in effect groups strains that may recombine more or less frequently, depending on their biology, as opposed to rare interspecies gene transfer. For bacteriophages, which show various lifestyles, the nature of the fundamental natural unit, if it exists in a biological sense, is not well understood and defined. The approaches based on dot-plots are useful to group similar bacteriophages, yet are not quantitative and use arbitrarily set cut-offs. Here, we focus on lytic *Myoviridae* and test the ANI metric for group delineation. We show that ANI-based groups are in agreement with the International Committee on Taxonomy of Viruses (ICTV) classification and already established dot-plot groups, which are occasionally further refined owing to higher resolution of ANI analysis. Furthermore, these groups are separated among themselves by clear ANI discontinuities. Their members readily exchange core genes with each other while they do not with bacteriophages of other ANI groups, not even with the most similar. Thus, ANI-delineated groups may represent the natural units in lytic *Myoviridae* evolution with features that resemble those encountered in bacterial species.

**Impact Statement**

While bacteriophage classification has traditionally relied on electron microscopy, the type of nucleic acids carried and the properties of bacteriophage tails, there is consensus that a whole bacteriophage genome should also be used in classification. This has been done at the subfamily and genus levels, yet on the finer scale, dot-plot approaches are mainly used. We introduce the average nucleotide identity (ANI) metric, which is widely used in bacterial species circumscription, for fine-scale lytic *Myoviridae* classification. We observe that these bacteriophages are grouped by ANI into discrete groups whose members exchange genes among themselves but not with members of other groups. Thus, ANI may possibly be a suitable classification metric for naturally occurring groups of lytic *Myoviridae*. As the latter are the main constituents of bacteriophage cocktails, which are promising alternative antimicrobial agents, ANI may also prove useful in their design as informed bacteriophage choice may delay or minimize the risk of resistance development.

## Introduction

In bacterial taxonomy, a species is proposed based on the monophyly, and genetic and phenotypic coherence of its members [1]. Although a continuum between species was suspected for a time [2] this remained an exception to the rule as shown in large-scale cultured strain genome comparisons [1]. There is a discrepancy, however, between the breadth of bacterial species from a taxonomic and ecological point of view because the former emphasizes the common evolutionary history and the latter the current niche adaptation, which may often circumscribe a narrower collection of strains [3]. Yet metagenomics studies have also shown that related bacteria from similar (interconnected) habitats and in similar living conditions form separated sequence clusters, possibly meaningful units of ecological diversity [4].

The genetic coherence of strains/sequence clusters is usually determined by the average nucleotide identity of the shared DNA (ANI), and for inclusion into species, ANI of the strains must be at least 96 % [1, 5]. A small (<5 %) but notable group of isolated strains show ANI in the range 93–96 %. They are considered genomovars, groups of strains that could be described as novel species depending on the ability of taxonomists to retrieve relevant phenotypic differences. Below this value there is a large discontinuity to average ANI of 84 % observed for different species of the same genus [1]. The main cohesive forces maintaining these observed sequence clusters/species with ANI above 96 % are thought to be intraspecies (cluster) gene recombination and periodic selection. Their contribution may vary drastically depending on the biology of particular species [6]. While some species such as *Helicobacter pylori* or *Shewanella baltica* are highly recombinogenic, others such as *Staphylococcus aureus* exchange genes among strains much less frequently, i.e. evolve more clonally [7–9]. Additionally, recombination is inhibited by sequence divergence, dropping 100-fold for sequences that are 10 % divergent [10].

The bacterial viruses, the bacteriophages, are classified into orders and families according to the nature of nucleic acids they carry and morphology. The majority (>95 %) are the *Caudovirales*, possessing heads filled with dsDNA and further divided into *Myoviridae*, *Siphoviridae* and *Podoviridae* depending on tail morphology and flexibility [11]. The family affiliation may also be complemented by bioinformatic analysis of their virion structural proteins [12]. Subfamilies and genera are defined from the whole bacteriophage genomes based on the proportion of shared proteins (as defined by blastp score of at least 75), the cut-offs being 40 and 20 % for inclusion within a genus and subfamily, respectively [13–15]. On the finer scale, bacteriophages are often compared using a dot-plot approach [16–18] where a syntenic similarity in over 50 % of their genomes was proposed as a cut-off for inclusion into ‘clusters’. Yet the degree of similarity that is required remains rather undefined and it is unknown whether clusters represent the result of some underlying biological principle. By contrast, the ANI metric, which emphasizes degree of similarity, has already been used in bacteriophage genome clustering [16, 17, 19]. These studies focused mainly on *Siphoviridae* found in *Mycobacterium* and *Gordonia* and the number of *Myoviridae* was small, 16 in total. Recently, two approaches to bacteriophage classification based on blast comparisons of bacteriophage genomes were published: vConTACT [20] is a re-evaluation of a gene-sharing network using more than 2000 bacteriophage genomes, while VICTOR [21] reconstructs phylogenetic trees using distance matrices obtained from transformed blast results.

The *Caudovirales* families are known for their contrasting lifestyles: *Siphoviridae* are usually temperate, residing in microbial cells for extended periods of time and prone to frequent lateral gene transfer leading to widespread mosaicism [22]. The *Myoviridae* on the other hand are predominantly lytic and may exchange genes much less frequently presumably only upon co-infection. At the time of writing this manuscript, a large-scale bacteriophage comparison study [23] was published that indeed corroborated this. By comparing over 2000 bacteriophages by an nucleotide word frequency (kmer) approach versus gene content it was shown that the analysed phages generally belong to either high or low gene content flux groups distinguished by their respective rates of lateral gene transfer. Lytic phages were shown to belong largely to the low gene content flux group. Thus, the observed lineages and clusters in tailed bacteriophage families are the result of different evolutionary scenarios that may produce different characteristics in bacteriophages leading disparate lifestyles. Here we extend the above findings of Mavrich and Hatful [23] and describe these characteristics for a sample of over 180 lytic *Myoviridae* bacteriophages infecting *Enterobacteriaceae*. We observe the distribution of ANI values while simultaneously taking into account genome coverage, which essentially incorporates the dot-plot approach information into analysis. We then evaluate the resulting groups relative to the existing classification and estimate the presence and extent of recombination taking place among members of these groups. We also test its extent relative to the lateral gene transfer from other groups. In effect, we demonstrate that lytic *Myoviridae* groups delineated by ANI may be reminiscent of bacterial species, in a sense that there is generally a large discontinuity among them and the gene pools are well separated.

## Methods

### Genomes and bioinformatic methods

Lytic *Myoviridae* bacteriophage genomes were obtained in 2016 from NCBI and EBI (accession numbers in Table S1, available in the online version of this article). ANI (blast algorithm ANIb, 500 bp fragment length) was computed using Jspecies [5]. For every genome pair Jspecies separated the query genome into 500 bp non-overlapping fragments, performed blast searches against target genomes for all of them and calculated average identity. ANIb coverage was obtained by a simple shell script using Jspecies blast results and validated against Jspecies coverage results; these are interactive, but hard to tabulate for all genome pairs. The pairwise homoplasy index [24] was used as the intragene recombination test and was run on PGAP-defined [25] and mafft-aligned [26] core genes of ANI-delineated bacteriophage groups. PGAP takes as input a complete set of predicted proteins and genes of all the genomes in a sample and performs an all-versus-all blastp search. The genes found in all genomes [using the gene family rule of 50 % amino acid identity (AAI) and at least 50 % coverage] make up the core genome. The neigbour-joining dendrogram of T4-like bacteriophage gene presence–absences was produced by PGAP. The phylogeny reconstruction based on (PGAP-defined) conserved genes of related ANI groups was done by PhyML as implemented in SeaView [27]. The phylogenetic congruence of core genes in ANI-delineated groups and of shared genes when comparing different groups was estimated using the congruence among distance matrices (CADM) test of the R package ape [28]. Multiple gene handling to produce gene distance matrices (model TN93) and reconstruction of phylogenetic trees for all the core or shared genes simultaneously was done using the R package apex [29]. The consensus net was reconstructed using neighbour-joining gene phylogenetic trees with SplitsTree4 [30]. The clonal frame in ANI groups was estimated using Gubbins [31] run on the core genome produced by Roary [32], a novel pipeline that is drastically faster than PGAP for core genome reconstruction due to filtering out almost identical sequences before the blastp stage. The gff files for Roary were produced from bacteriophage nucleotide sequences using Prokka [33], a customizable local annotation pipeline.

## Results

### ANIb and coverage

**Fig. 1.**
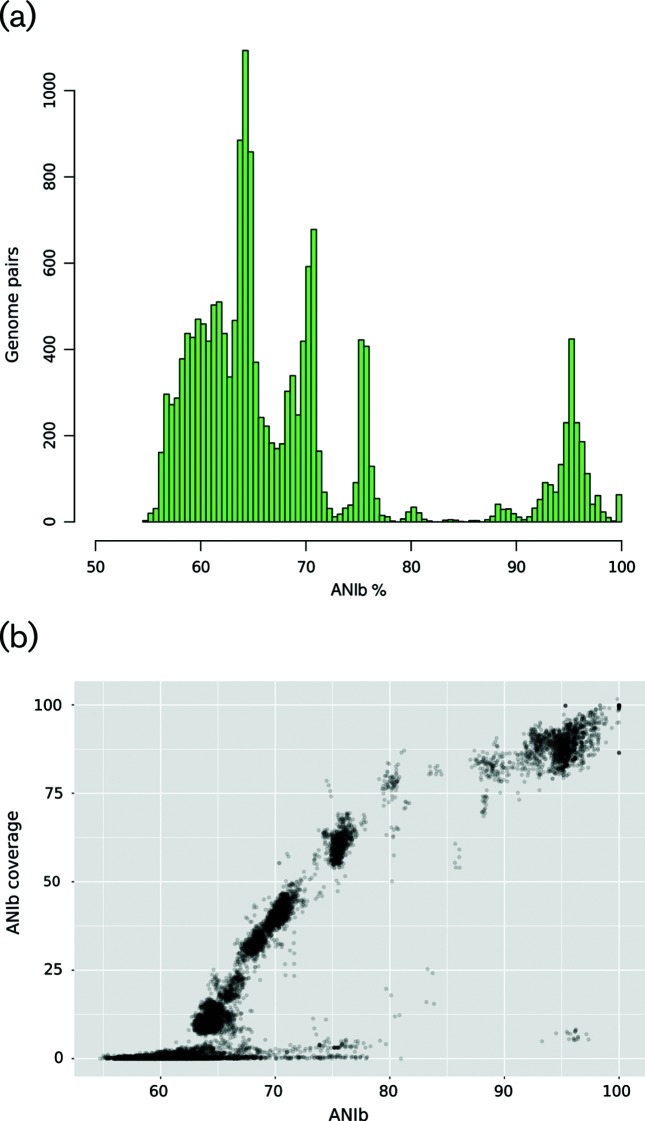
ANI analysis of the 186 lytic *Myovridae* bacteriophages. (a) Histogram of ANI values showing a discontinuity centred on 85 %. (b) ANIb versus ANIb coverage (the proportion of the bacteriophage genome on which the ANI was actually calculated, the shared DNA).

A comparison of all-versus-all 186 lytic *Myoviridae* bacteriophages using ANIb can be seen in Fig. 1(a) and Table S2. There is a clear discontinuity between highly similar bacteriophage genomes centred at around 95 % ANI and the majority of all other pairs whose ANI is less than 80 %. There are also some pairs that exhibit ANI in the range 85–91 %, and we term these satellites. Because high similarity could be confined to a small portion of genomes, introducing added complexity to ANI analysis as noted earlier [17] for *Siphoviridae*, we also measured the portions of the genomes on which ANI measurements were actually made and these are presented in Fig. 1(b). It is clear that the vast majority of highly similar genomes also share at least 80 % of DNA and thus form groups of bacteriophages with highly similar gene content. Based on a 92 % cut-off, a non-arbitrary boundary of highly similar genomes, we delineated groups of bacteriophages (Table S1).

The groups are in agreement with earlier studies [34] and the dot-plot clusters [16] with the added refinement of cluster I of T4-like phages, which according to ANIb is split into groups KP15 and RB43, the latter surrounded also by ‘satellite’ RB16, Lw1 and GAP161 bacteriophages. A similar pattern can be seen for cluster C, where dot-plot suggests homogeneity [16] yet ANIb clearly separates the JS10 and JS98 groups from bacteriophages of the IME08 group (whose members are themselves barely over the 92 % cut-off) and their satellites. Interestingly, groups JS10 and IME08 are themselves close, suggesting the recent divergence of these bacteriophages. Thus, ANIb being quantitative may give clearer results than dot-plot in these cases. The ANI-delineated groups are also in agreement with the International Committee on Taxonomy of Viruses (ICTV) classification (ICTV master species list 2016 v1.3) and all of the groups contain only one genus classification (Table S1). In several cases, the ICTV genus is split among two or even three ANIb groups. The genus *Vi1virus*, for example, is split into groups Marshall and Phax (‘satellite’ relationship at 88 % ANIb) and Limestone (73 % ANIb). Other examples may be found in the above-mentioned genus *Js98virus* and the genera *Se1virus, Cr3virus* and *Msw3virus*.

All of the bacteriophage pairs that produced other notable ‘islands’ (Fig. 1b), with lower ANIb values down to the 63 % and coverage over 12 %, are members of the same ICTV subfamilies, but different genera within *Myoviridae.* For the T4 group, for example, the island around 73–78 % ANIb corresponds to *T4virus* vs *RB69virus* comparisons, while that of 67–72 % corresponds to *T4virus* vs *JS98virus* comparisons. The more distant *RB49virus* genus is still above the noise at around 64 % ANIb and 15 % coverage, but members of different subfamilies (e.g. Felix of *Ounavirinae*) are too divergent to be detected using the ANIb metric. The conspicuous comparisons with low coverage and high ANIb (around 95 %) involve all of the phages from the RB69 group and the *Escherichia* phage Av-05 in which horizontal transfer of almost 10 kb of contiguous sequence probably occurred. This sequence corresponds to the T4 genes from *rI* to *e.2*.

As a control to ANI calculations, we reconstructed a phylogeny of the T4-like bacteriophages [16] based on 27 conserved genes, which produced the same groups as the ANIb delineation, with again the recent divergence in the JS10/IME08 groups (Fig. S1a, genes in Table S3). Additionally, the gene content dendrogram provided by PGAP analysis (Fig. S1b) supports the same groups. Interestingly, the T4 bacteriophage may not be the most typical of its group.

### Recombination in ANIb-delineated group members

The pairwise homoplasy (phi) test based on (in)compatible site analysis of aligned alleles of shared genes was used to test for recombination. Rrecombination was detected readily in many core genes of the more populous groups (Table 1). As noted previously, [24] this test is conservative when sequence diversity is low and when there are few samples, which could partially explain the variability observed. The extent of recombination taking place among related strains may also be inferred from determination of the clonal frame, which is the portion of the sequence that has not experienced recombination since the isolates diverged from their most recent common ancestor. We used Gubbins [31], which implements an SNP density approach to discern regions with elevated SNP numbers indicative of recombination. The ranges of clonal frame of all phylogenetic tree branches of bacteriophage groups are also given in Table 1 and confirm the widespread presence of recombination detected by the phi test.

**Table 1.** Evidence for recombination in members of the more populous ANIb-delineated bacteriophage groups The CADM test measures the similarity among distance matrices obtained for all core genes of a sample. It is given as the Kendall coefficient, where 1 indicates complete agreement among distance matrices of all genes and 0 the opposite. A low *P*-value indicates that at least two matrices are similar and the total incongruence is rejected.

Group name (number of phages)	All genes (50 % AAI, 50 % coverage)	Core genome	Genes exhibiting recombination (pairwise homoplasy test)	Clonal frame, portion of core DNA (range for all tree branches)	CADM, all core genes W, *P*-value
T4 (32)	434	183	123	0.86–0.98	0.16, *P*<0.001
Felix (17)	224	88	48	0.80–0.97	0.27, *P*<0.001
FV3 (11)	314	175	74	0.81–0.94	0.32, *P*<0.001
RB69 (9)	334	203	73	0.90–0.97	0.12, *P*<0.001
Phax (9)	383	135	55	0.71–0.91	0.06, *P*<0.001
KP15 (6)	335	207	40	0.89–0.96	0.09, *P*<0.001
Ea35-70 (5)	341	307	55	0.93–0.97	0.03, *P*<0.001
Marshall (5)	268	148	17	0.66–0.88	0.18, *P*<0.001

**Fig. 2.**
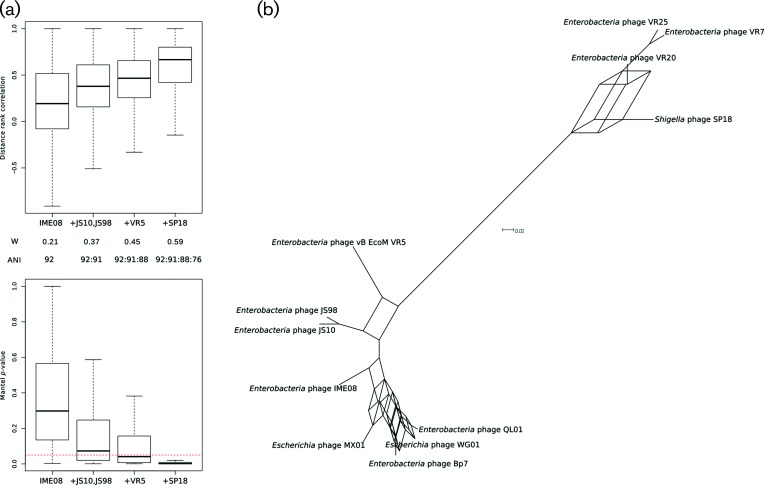
The phylogenetic congruence in samples containing bacteriophages of different ANI-delineated groups and putatively recently diverged bacteriophages (‘satellites’). The groups and bacteriophages were chosen to cover the transition zone from highly similar genomes to clearly separated groups displaying 75 % ANI. Starting with the group IME08, successive designated satellite phages and finally whole SP18 group phages were added. (a) The CADM test applied to shared genes of each sample. W: the Kendall coefficient of concordance among gene distance matrices. W may range from 1 (distance matrices for every gene are the same) to 0 (no similarity in matrices). The boxplots above represent Mantel correlations among rank-transformed distance matrices for all shared genes. By adding progressively more distant bacteriophages, the distance matrices are becoming more similar, i.e. the same bacteriophage clusters are recorded in more and more matrices/genes. Below is the significance of the correlations where the red dotted line indicates *P*=0.05. For sample IME08, most matrix correlations are expected to arise by chance, while for the sample containing the SP18 group, the correlations are significant. (b) Consensus network constructed for a sample containing all of the bacteriophages in (a), illustrating the CADM results. There is clear clustering between ANIb-delineated groups but a relative lack of it at the intragroup level.

Frequent recombination events taking place among members of a bacterial population lead to variation in the evolutionary history of analysed genes found in a specific member: that is, the gene phylogenetic reconstructions may produce different trees [35–37]. On the other hand, lateral gene transfer among bacterial species is not as frequent and the phylogenetic reconstructions of housekeeping genes are congruent between the species [38]. We used CADM to test observations made in bacteria in our bacteriophage sample. This indicated that while there is almost no or only limited agreement of the distance matrices of the core genes among members of the ANIb-delineated groups, at least some of them may be congruent, as evidenced by the *P*-value (Table 1). The a posteriori CADM Mantel tests [39] confirmed that intragroup correlations among matrices were weak and the probability of observing them by chance was high (Fig. S2). Next, we applied the CADM test to samples where in addition to the starting ANI group, the progressively more divergent bacteriophages (‘satellite’) and/or whole related bacteriophage groups were added. The congruence among distance matrices and their individual correlations increased when ANI decreased and, at the same time, the correlations were more significant until upon addition of divergent phages (Figs 2a and S3a, b) most or nearly all matrices were similar and not expected to arise by chance.

To illustrate the incongruity of the gene phylogenetic reconstructions obtained using the above gene distance matrices, consensus networks [40], a generalization of the consensus tree approach, were used. They graphically represent the splits among taxa seen in input trees and may represent conflicting tree topologies; the split weights here are proportional to the frequency of the split’s occurrence in the data [41]. The consensus network in Fig. 2(b) shows the conflicting tree topologies among the members of the same ANIb-delineated group (SP18 and IME08) while at the same time the groups are clearly separated by only one split, demonstrating that members of different groups are almost always separated in individual gene trees. Further examples of intra- and intergroup relationships straddling the ANI range 75–94 % can be seen in Fig. S3(c), confirming this result.

We also investigated what the actual differences among ANIb-delineated groups may be in terms of gene content. First, the core genome of the 32 members of the T4 group was constructed (182 genes) and then genes common with the next most similar group, RB69, were determined. The difference was in genes putatively associated with group-specific markers, which are shown in Table S4. Beside many genes with products of unknown function, there were also *42, a-gt, rIIA* and *Imm.1*.

## Discussion

ANI proved to be a suitable metric to classify the lytic *Myoviridae* bacteriophages for several resons: it is well correlated to gene content, is quantitative and, while in agreement with ICTV classification of genera and dot-plot groups, has greater resolution than the latter. It also appears to delineate naturally occurring lytic *Myoviridae* bacteriophage groups. The kmer nucleotide word frequency approach may also be used as a faster means to calculate proximity compared with ANI [23] with slightly less resolution and range, yet ANI is also not usable below approximately 63 % (Fig. 1). The curve in Fig. 1(b) seemingly flattens at values above 80 % ANIb, indicating the same shared DNA proportions for phages above this value. This suggests that the initial isolation of *Myoviridae* bacteriophage lineages mainly involves nucleotide divergence of existing genes and not the lateral gene transfer of the longer (frequently core) genes. This is not observed in the lytic–lytic kmer frequency vs gene similarity comparison in the study of Mavrich [23]. Several reasons may be proposed to explain this. First, they used the gene content index while we plot the proportion of shared DNA, and the genes have different lengths. Secondly, the sample used was not homogeneous in the lytic–lytic comparison as bacteriophages from 158 *Myoviridae*, 85 *Podoviridae* and 204 *Siphoviridae* were used [23], effectively summing the possible differences in various bacteriophage family backgrounds. Thirdly, the slightly reduced resolution of the kmer approach may ‘compress’ the kmer-derived nucleotide distance relative to ANI as evidenced in Fig. S2(d) of the Mavrich study. Lastly, even bacteriophages of the *Enterobacteriaceae* may not have been sampled thoroughly as evidenced by 30 singleton bacteriophages (Table S1). Therefore the shape of the curve in Fig. 1(b), though likely, is not conclusive and some bacteriophages analysed here (coverage of 52–60 % at 86 % ANI) may in fact follow the high gene content flux.

The above differences aside, we focused on the properties of observed highly related bacteriophage groups. Several lines of evidence suggest that they represent naturally occurring groups: there is a discontinuity in the ANI histogram much like the one seen in *Bacteria*, which separates highly similar bacteriophages from those in other groups, suggesting long-term stability of only more gene-divergent lineages, which may escape the recombination cohesion. Recombination between members of one group is in fact readily detected using different approaches and its consequences are apparent in the phylogenetic incongruence of the core genes. Yet when several groups are examined for phylogenetic congruence at the same time, a clear group clustering is seen, suggesting the genes are exchanged and fixed among groups only rarely and the groups possess separate gene pools. This has been shown for two representatives of two related ANIb groups, bacteriophages T4 and RB69, as they produce no recombinants and RB69 actually excludes T4 [34]. The notable gene content differences among the T4 and RB69 groups were assessed and the prominent gene products present in the T4 group but not in RB69 were α-glucosyl transferase, dCMP hydroxymethylase, *rIIA* product and Imm.1. The first two were envisaged by Petrov *et al*. [34] as possible drivers of bacteriophage genetic isolation as they lead to preferential propagation in strains with distinct restriction systems and also open possibilities for nucleases to act differentially on DNA with various modifications. The latter two, being involved in superinfection exclusion, may selectively inhibit some bacteriophages but not others, but this remains to be proven. In addition, genes directing host RNA polymerase to use only modified DNA (*alc*) were proposed to have a role in genetic isolation [34]. Interestingly, *modAB*, *mrh* and *srh*, found in all members of the T4 group but not in the RB69 group, manipulate transcription but these genes are not known to possess function such as *alc*. Further examples of genes contributing to the genetic isolation of these groups may also be uncovered among functionally uncharacterized genes (Table S4), which are conserved over 32 bacteriophages of the T4 group.

In conclusion, the lytic *Myoviridae* analysed display several features reminiscent of bacterial evolution whereas for the temperate bacteriophages alternative classification schemes may be needed. In addition, the findings presented here should be extended to lytic bacteriophages of other bacterial groups to verify whether they pertain to the lytic *Myoviridae* in general. Preliminary evidence for *Campylobacter* phages suggests this may be true.

## Supplementary Data

1285 Supplementary File 1

## Supplementary Data

208 Supplementary File 2
