# Supplementary material for: The lytic Myoviridae of Enterobacteriaceae form tight recombining assemblages separated by discontinuities in genome average nucleotide identity and lateral gene flow
Source: Microb Genom. 2018 Mar 27;4(3):e000169. doi: 10.1099/mgen.0.000169 (PMC5885020; doi:10.1099/mgen.0.000169)
Supplement: Supplementary File 1 [file mgen-4-169-s001.pdf]

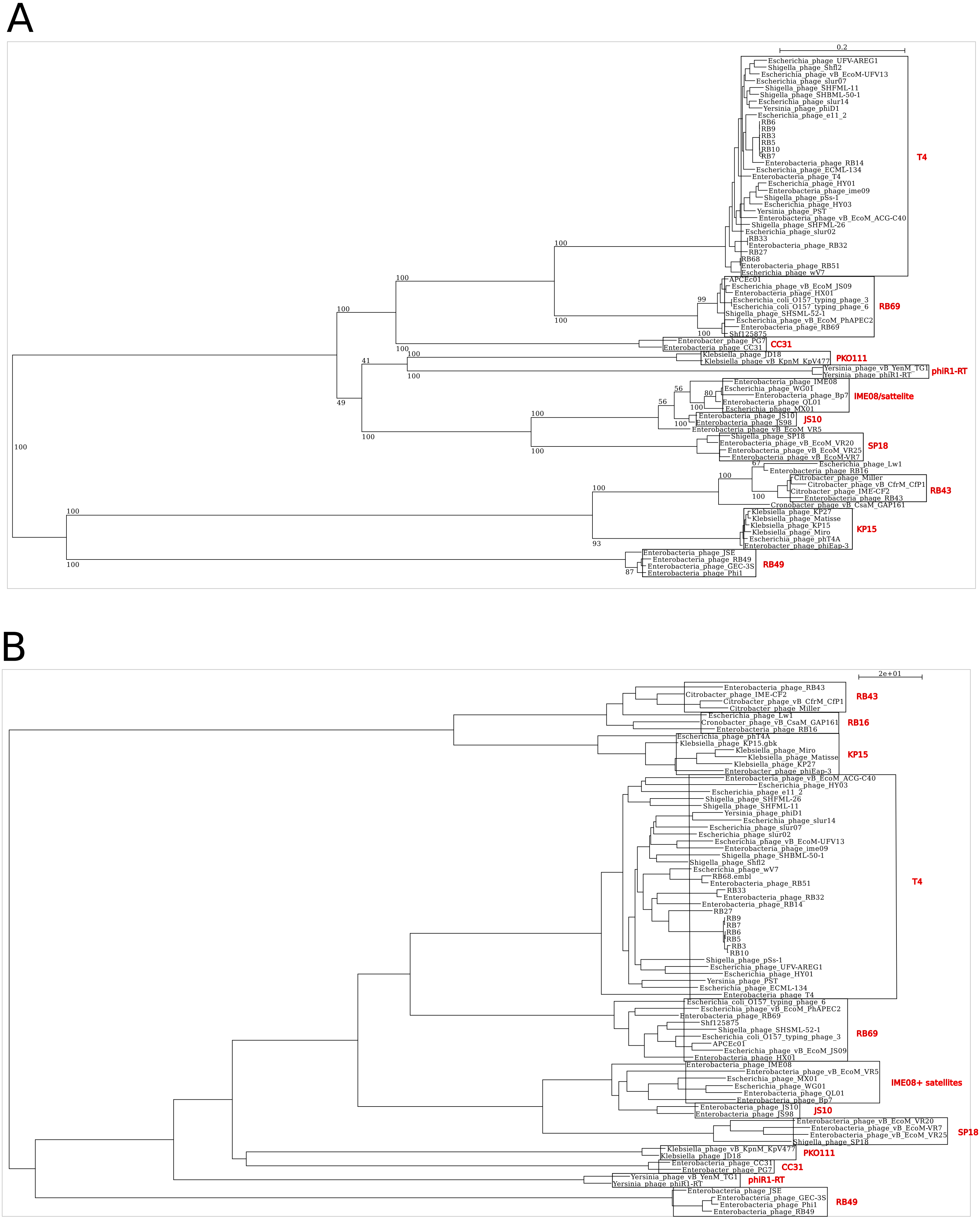

Figure S1. A: core genome phylogeny reconstruction of T4-like bacteriophages. B: gene content similarity dendrogram of the same bacteriophages based on a gene family rule (at least 50 % AA identity over at least 50 %).

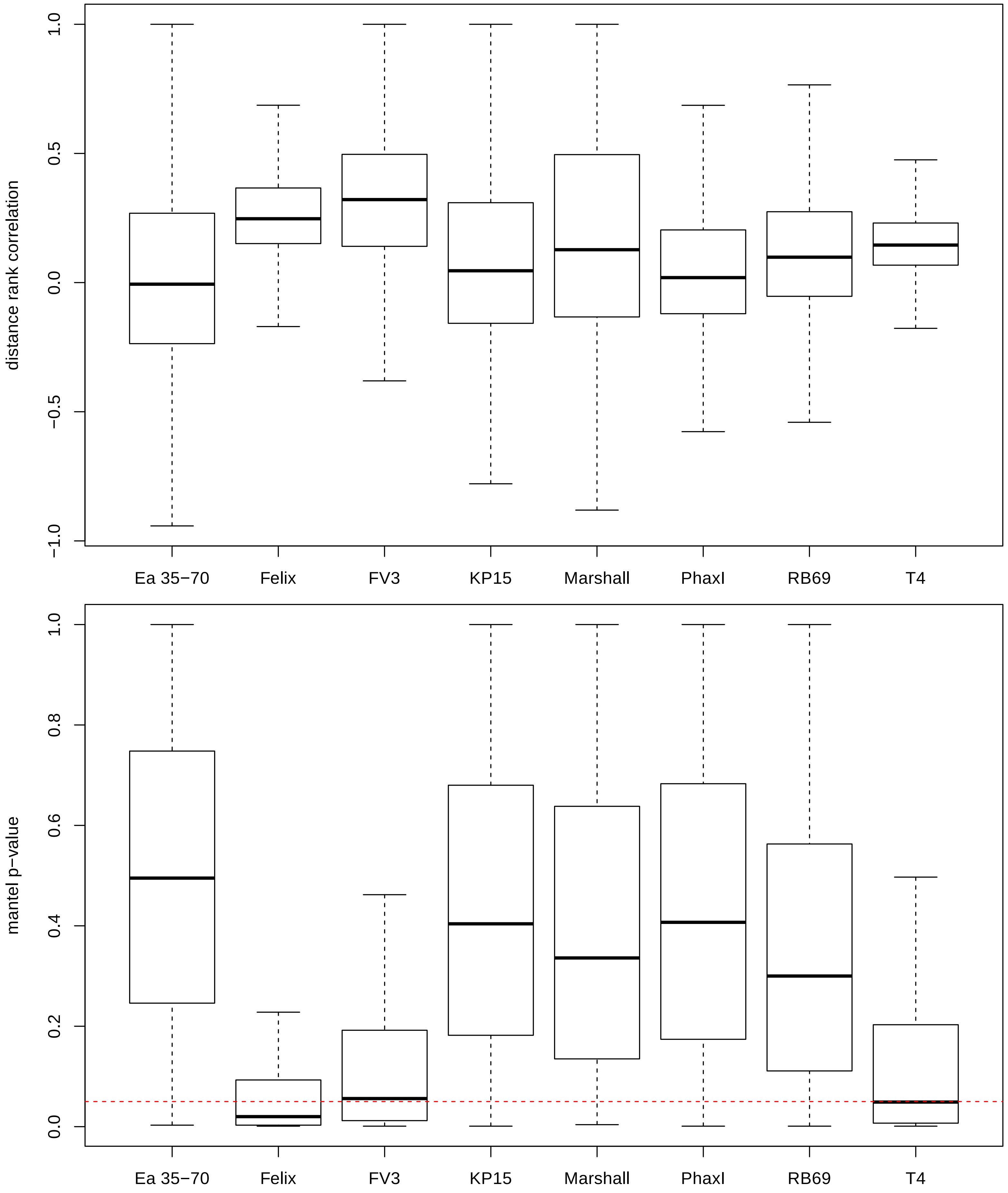

Figure S2. The correlations between all core gene distance matrices and their significance in ANI delineated groups.

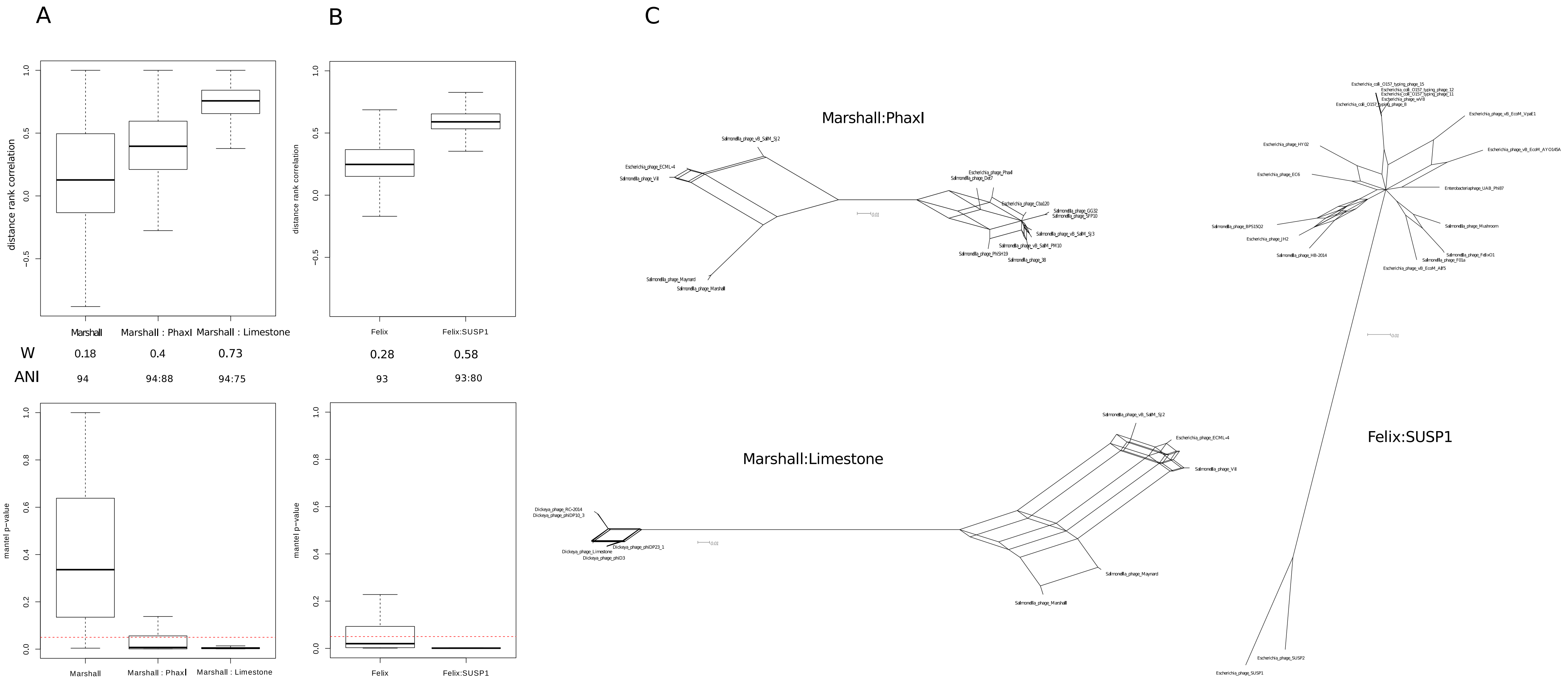

Figure S3. Left: Congruence of distance matrices analysis of the core genes. A, B: comparison of bacteriophages from groups (A) Marshal vs PhaxI and Limestone and (B) Felix vs SUSP1. Above are matrix distance correlations, below corresponding probabilites. C: Consensus network diagrams for these comparisons.
